# It Is Not Enough to Assess Conflicts of Interest When We Bring the Commercial Sector to the Policy Table Comment on "Towards Preventing and Managing Conflict of Interest in Nutrition Policy? An Analysis of Submissions to a Consultation on a Draft WHO Tool"

**DOI:** 10.34172/ijhpm.2021.148

**Published:** 2021-10-25

**Authors:** Elisa Chilet-Rosell, Ildefonso Hernández-Aguado

**Affiliations:** ^1^Department of Public Health, Universidad Miguel Hernández, Alicante, Spain.; ^2^CIBER de Epidemiología y Salud Pública (CIBERESP), Madrid, Spain.

**Keywords:** Conflict of Interest, Undue Influences, Governance, Nutrition, Policy-Making

## Abstract

Ralston et al offer us an interesting analysis of the consultation process of World Health Organization’s (WHO’s) "Draft approach on the prevention and management of conflicts of interests in the policy development and implementation of nutrition programs at country level," in which it shows us how the industry tries to frame the discussion in individual conflicts of interest, avoiding structural conflicts of interest. We must not forget other issues of importance in policy-making, such as the imbalance of power between different actors and the strategies of undue influence used by food and beverage corporations. It is essential to develop regulatory-based tools and procedures that embody ethics and good governance and that can be applied systematically and routinely to prevent corporate influence in health policy-making. A global observatory of corporate practices would also be needed to recommend to governments efficient actions to avoid corporate capture of their policies.

## Introduction

The article by Ralston et al^1^ carries out an interesting analysis on how different actors conceptualize conflicts of interest, based on 44 responses to the online consultation process of the “Draft approach on the prevention and management of conflicts of interests in the policy development and implementation of nutrition programs at country level” (hereafter WHO tool).^2^ The fact that numerous commercial sector entities have participated in the consultation process points to the importance of this tool (14 commercial actors, 12 from non-governmental organizations [NGOs], 8 from academic institutions and 6 from Member states). Although neutral, Ralston et al clearly show us the intention of the commercial sector entities to frame de discussion of the conflicts of interest to a narrow and circumscribed view to individual-level conception as well as their influence in the elaboration and implementation of policies.

 In this commentary, we broaden the discussion with other issues in policy-making, such as the imbalance of power between different actors and the public response to the strategies of undue influence in the policy-making process that transnational food and beverage companies use to avoid effective nutrition policies.

## The Importance of the Framework in Health Policy

Framing defines the problem in a certain way and anticipates a type of solution.^3,4^ For example, obesity is usually presented as a matter of individual free choice while the fundamental role played by obesogenic environments is almost absent from public space.^3,5-8^ The role of government and regulation is, therefore, limited. This framework leads the resolution of the problem to be entrusted to the private sector and self-regulation measures. Presenting obesity as a matter of individual responsibility results in inadequate public health responses, and this prevents the control of a problem in which environmental and political causes play an important role.^6^ Individual focus is again invoked by the commercial sector entities in the World Health Organization (WHO) tool consultation process.^1^ Given the distinction made by WHO on individual and institutional conflict of interest, the commercial sector entities focus on personal conflict of interest, thus simple solutions such as the disclosure of interest at the individual level are the preferred solution. Disclosure makes explicit and transparent details that are important to the interpretation and credibility of the information presented. However, focus on disclosure of conflict of interest can leave unaddressed other aspects that need to be considered when collaborating with other actors and mask potential conflicts of interest for commercial actor. The arguments of NGOs and some Member States focus on the structural definition of conflict of interest – more difficult to manage- and provide examples in which these have undermined public health objectives; thus, showing their concerns regarding the participation of corporate entities in the elaboration of policies and even demanding their non-inclusion. Even though these arguments are based in evidence, in global health discussions the predominant framework is more favourable to establish collaborations with corporate entities. The private sector has managed to present a scenario in which it shows itself as a valid political actor rather than the opponent or the receiver of nutrition policies.

Despite the fact that there is still a lack of sound evidence supporting the effectiveness of public-private partnerships in health promotion,^1,9^ these engagements have been widely recommended as part of the solution to address global health challenges, as reflected in the Sustainable Development Goals. Even more, the global agenda is used to discredit the WHO tool in the analysis of Ralston et al^1^ and have been described as an obstacle.

## Imbalance of Opportunities and Resources

 In our view, there has been an enthusiastic acceptance of public-private partnerships for public health policy development without parallel design of good governance mechanisms to avoid power imbalances towards actors with more capacity and influence on other actors and on governments and multilateral institutions. The push for public-private partnerships has meant that we are very busy reducing harm damage, while the actors with the most resources devise strategies to influence public health policies that we are not yet able to envision. Damage reduction tasks in public-private partnerships include managing conflicts of interest.

 The analysis of Ralston et al included the importance given by some Member States (Colombia and Namibia) to the WHO’s tool to protect the vulnerable against conflicts of interest in the nutrition policy-making process. However, it is important to highlight that global health and health policy must consider the distribution of power at a global level and within countries. In this discussion Member States should pay also attention not only to conflict of interest but also to imbalances of power between different actors. This power imbalance between public health advocates and nutrition large corporations was described in the debate in the European Parliament on the front-of-pack ‘traffic light’ system, which was defended by different NGOs, versus a system based on guideline daily amounts -defended by the Confederation of the Food and Drink Industries of the European Union (CIAA).

Nutrition commercial entities are aware of the importance of establishing a framework in political decision-making processes, and therefore allocate large amounts of money to favour their position in the debate. CIAA spent 1 billion euros opposing proposals for front-of-pack ‘traffic light’ labels in favour of a system based on guideline daily.^10^ Kurzer and Cooper analysed this debate.^10^ According to them, during the draft phase, the NGOs succeeded in framing the debate in favour of traffic light labelling as a tool to improve public health and fight obesity. However, this dominant position changed during the process due to the imbalance of power between the NGOs and industry, whose activities were implemented at all European Union power levels. At the end of the debate, the conclusions supported the arguments of industry. To date, industries have had more opportunities and resources than other sectors of the population when influencing decision-making bodies. Not only do corporations have more power, they may use it unfairly with the aim to derail and delay policies that may harm their interests, including at the WHO.^11^

## Strategies of Undue Influence in the Policy-Making Process

In the WHO tool consultation process, the collaboration and partnership frame, which was mainly proposed by nutrition sector entities, claimed that it cannot be compared to the tobacco industry. However, both sectors have shared strategies to influence public policies that could harm their image and interests. As described in “The Corporate Playbook, Health, and Democracy: The Snack Food and Beverage industry’s Tactics in Context,” some food and beverage corporations have used tactics that discredit public health actions, such as distorting scientific information and using financial tactics and political influence to avoid unfavourable regulations.^12^

The food industry is often described as having more influence in nutrition policymaking than nutrition professionals, scientists and other practitioners working for the public interest.^13^ As we previously described from interviews with key informants in the case of policy-making process in Spain, the private sector has a greater information capacity than the government regarding both technical and strategic information.^14^ This imbalance favours interest of private sector. Key informants stated that the companies had access to the agendas of internal government meetings and also to the content and the positions of different members. This information was used to design strategies of influence.

 In these interviews, the informants stated that commercial sector uses different strategies that range from subtle influences to overt corruption. One of the main factors contributing to the success of these undue influences, particularly subtle ones, is the lack of sufficient technical and strategic capacity in the public sector. In this situation, a new political practice by which policy decisions of the cabinets are more in line with the media agenda than the political program of the government has been gaining ground. This media agenda is easily formatted by the influence and power of industry and creates a context where policies are designed to gain media attention and popularity.

## Involving Commercial Entities in Public Health Nutrition Policies Making?

Despite the interest shown by public-private collaboration in public health, evidence shows that public private partnerships have limited effectiveness and that there is also a real conflict between the role of public health as social good and profit-driven agendas.^15^ WHO tool could reinforce institutional procedures and structures to guide nutrition policy-making when it includes the participation of the commercial sector.

 However, it is important to define the terms and parameters of an appropriate engagement with non-state actors in policy-making. In addition, it is also necessary to design and implement procedures that combine ethical and good governance issues, which can help to create a lasting institutional culture which will prevent interactions that may harm public health. We need clear and effective institutional policies that put the public interest at the centre of nutrition policy-making. This process should respect the principle of equity, which means that population groups representing the more disadvantaged sectors should be given more opportunities to express their positions and views. In this sense, WHO’s consultation process should also have included the principle of equity. As Ralston et al highlighted, the consultation process was dominated by the private sector and high- middle and high-income countries. WHO’s consultation process should have facilitated the participation of low- and lower-middle income countries and non-English speaking countries, as well as other civil society sectors. As Ralson et al pointed out in their paper, WHO tool was presented as “a living document to be revised.” In this revision, voices and experiences of frequently under-represented sectors and countries should be prioritised.

## Conclusion

The current context favours the participation of the commercial sector in the elaboration of policies. However, we must be aware and vigilant of potential risks. In this sense, the WHO tool is useful for evaluating potential conflicts of interest that can guide decision-making and help to identify specific actors and forms of engagement in situations where conflict of interest can be managed to protect public health goals. An important aspect of the WHO tool identified by Ralston et al is that it helps member states to move beyond a binary approach to industry engagement: partnership or exclusion. Ralston et al have shown that conflicts of interest are central to debates around the role of the commercial sector in nutrition policy-making: Also, they have shown that the way in which conflicts of interest are framed has consequences for the management of a successful collaboration free of undue influences. No doubt that governments must incorporate this tool and other useful proposals^16^ in the development of public policies on nutrition. However, interaction with corporations whose products are harmful for health is a wicked issue that requires strong policy capacity,^17^ ie, the ability to take and develop policy decisions in order to ensure that any public interaction with private actors has benefits for the health of the population and that there are no better alternatives to achieve the same goals.

 Policy capacity applied to the management of public private interactions to promote health require:

- Expertise and capacity to understand and evaluate the potential benefits of the interaction as well as its unintended consequences.
- Intelligence on process in terms of development of tools and procedures that embody ethics and good governance in administrative performance and are applied systematically and routinely by officials to public health interventions.
- Reduction of the power imbalance among stakeholders, so that the most vulnerable and least resourced stakeholders have a participation of a magnitude that compensates for their disadvantage.
- Expertise and competence in governments to pass appropriate legislation to improve the health of populations and to anticipate and defence against legal challenges.

Beyond policy capabilities, although closely related to it, research and training are key pillars to prevent the capture of public health policies by interests unrelated to the health of the population. The extraordinary capacity for innovation of transnational corporations warrants constant research on the practices that commercial sectors use to undermine public health. Perhaps a global observatory on commercial health determinants is needed. Training in ethics and good governance for health professionals and public officials in general is also essential.

## Acknowledgements

 The authors thank Jessica Gorlin for language editing.

## Ethical issues

 Not applicable.

## Competing interests

 Authors declares that they have no competing interests.

## Authors’ contributions

 ECR drafted the manuscript. IHA have made a critical revision of the manuscript for important intellectual content and agreed to the published version of the manuscript.

## Authors’ affiliations

^1^Department of Public Health, Universidad Miguel Hernández, Alicante, Spain. ^2^CIBER de Epidemiología y Salud Pública (CIBERESP), Madrid, Spain.
